# Role of AcsR in expression of the acetyl-CoA synthetase gene in *Vibrio vulnificus*

**DOI:** 10.1186/s12866-015-0418-4

**Published:** 2015-04-12

**Authors:** Min Jung Kim, Juri Kim, Hye Yeon Lee, Hyeon Jin Noh, Kyu-Ho Lee, Soon-Jung Park

**Affiliations:** Department of Environmental Medical Biology and Institute of Tropical Medicine, Brain Korea 21 PLUS Project for Medical Science, Yonsei University, Seoul, 120-752 South Korea; Department of Life Science, Sogang University, Seoul, 121-742 South Korea

**Keywords:** *Vibrio vulnificus*, Acetate metabolism, Acetyl-CoA synthetase, AcsR, VarS/VarA

## Abstract

**Background:**

VarS/VarA is one of the global factors regulating diverse aspects of the metabolism and virulence of bacteria including pathogenic *Vibrio* spp. An experiment to identify the VarS/VarA-regulon in *V. vulnificus* revealed that a putative LuxR-type transcriptional regulator was down-regulated in Δ*varA* mutant. To investigate the roles of this regulatory cascade, the target gene regulated by a LuxR-regulator was identified and its expression was characterized.

**Results:**

Transcriptomic analysis of the mutant deficient in this LuxR-type regulator showed that the *acsA* gene encoding acetyl-CoA synthetase was down-regulated. Thus, this regulator was named AcsR for “regulator of acetyl-CoA synthetase”. A putative histidine kinase gene, *acsS,* was located five ORFs downstream of the *acsR* gene. Expression of an *acsA::luxAB* transcriptional fusion was decreased in both Δ*acsR* and Δ*acsS* mutants. Similar to a Δ*acsA* mutant, strains carrying deletions either in *acsR* or *acsS* grew slowly than wild type in a minimal medium with acetate as a sole carbon source. Growth defect of the Δ*acsR* strain in acetate-minimal medium was restored by complementation. To investigate if AcsR directly regulates *acsA* expression, *in vitro*-gel shift assays were performed using the recombinant AcsR and the regulatory region of the *acsA* gene, showing that AcsR specifically bound the upstream region of the *acsA* ORF.

**Conclusion:**

This study indicates that the VarS/VarA system plays a role in *V. vulnificus* metabolism via regulating AcsR, which in turn controls acetate metabolism by activating the transcription of the acetyl-CoA synthetase gene.

**Electronic supplementary material:**

The online version of this article (doi:10.1186/s12866-015-0418-4) contains supplementary material, which is available to authorized users.

## Background

*Vibrio vulnificus* is a halophilic marine microorganism that is frequently associated with gastroenteritis and septicemia in humans with risk factors such as uremia and liver diseases [1]. The following microbial components of *V. vulnificus* have been determined as virulence factors: capsular polysaccharides [2], a cytolytic VvhA hemolysin [3], a contact-dependent RtxA toxin [4,5], an elastolytic VvpE protease [6], lipopolysaccharides [7], and a phospholipase A_2_ [8]. In addition to these extracellular components, any microbial factor enhancing growth or survival of *V. vulnificus* under diverse environmental conditions, such as iron acquisition [9], motility [10,11], and fermentation efficiency [12], is critical for its pathogenicity.

VarA had initially been discovered as a response regulator of the two-component family modulating virulence of *V. cholera* [13]. VarS was thought as a cognate histidine sensor kinase for VarA based on identification of BarA/UvrY, a VarS/VarA homologue of *Escherichia coli* [14]. VarS/VarA homologous systems are also present in other Gram-negative bacteria, which are differently annotated as BarA/SirA (*Salmonella*), GacS/GacA (*Pseudomonas*) and LetS/LetA (*Legionella pneumophila*) [15-17]. This two-component regulatory system plays a pleiotropic role in the signaling cascades for bacterial survival as well as bacterial pathogenicity upon reception of appropriate signals [18]. Well-characterized target genes of these VarS/VarA homologous systems include *csrB*- and *csrC*-encoding small RNAs (sRNA), the expression of which is positively regulated by VarS/VarA [19]. These sRNAs then sequester a regulatory protein, CsrA that directly controls the expression of several genes at post-transcriptional level [20].

In *V. cholerae*, VarS/VarA system is known to control the expression of HapA, a hemagglutinin/protease along with CsrA/*csrB*/*csrC*/*csrD* [21]. VarS/VarA also modulates expression of virulence proteins such as cholera toxin and toxin-coregulated pili by controlling ToxT expression [22]. In addition, VarS/VarA-CsrA/*csrB*/*csrC*/*csrD* system regulates quorum sensing in *V. cholerae* by altering the expression level of HapR, a master regulator of quorum sensing [23,24].

Little is known about VarS/VarA and CsrA/*csrB*/*csrC* systems in *V. vulnificus*. Quantitative measurement of transcripts in the *ΔvarA* mutant *V. vulnificus* demonstrated that the amount of sRNAs, such as *csrB1*, *csrB2*, *csrB3*, and *csrC* was reduced in the mutant as well as mRNAs encoding flagellins, RpoS, RtxA1, and VvpE [25]. Comparison of bacterial ability to form biofilm between *csrA*-positive and *csrA*-negative *V. vulnificus* strains clearly indicates that CsrA inhibits biofilm formation by *V. vulnificus* [26].

Based on the hypothesis that VarS/VarA could control other regulatory proteins in addition to the *csrB* and *csrC* sRNAs, we further searched VarS/VarA-target genes with special attention to any transcription factors. Among the down-expressed proteins in *ΔvarA* mutant, a putative transcription regulator with a LuxR-type DNA binding domain was selected and used to identify its regulon via comparative transcriptome analyses. Interestingly, expression of the acetyl-CoA synthetase gene (*acsA*) among others, was found to be reduced in a mutant defective in the LuxR-type regulator.

Production of acetyl-CoA occurs via two different catalytic reactions: i) Acetyl-CoA synthetase (Acs) forms acetyl-CoA from acetate through an acetyladenylate intermediate. ii) Alternatively, acetyl-CoA is formed via two enzymatic reactions catalyzed by acetate kinase (Ack) and phosphotransacetylase (Pta). In *E. coli*, Acs activity is induced by acetate and repressed by glucose. Thus, Acs functions as a high-affinity acetate uptake system scavenging extracellular acetate present at relatively low concentration [27]. On the other hand, Ack and Pta primarily play a catabolic role showing a low affinity toward acetate. Although these two catalytic reactions appear to be present in *V. vulnificus* based upon genomic sequence analysis, which shows the presence of *acsA* (VVMO6_00187) and *ack* (VVMO6_01096)/*pta* (VVMO6_01095), little information regarding the functions and expressions of acetyl-CoA synthesizing enzymes is available in this species.

In the present study, the *acsA* gene was chosen from a series of comparative analyses of gene expression using DNA microarrays, and the regulatory mechanisms for *acsA* expression were examined.

## Results

### Effect of the *varA* mutation on expression of various transcription factors, including a LuxR-type regulator

The VarS/VarA two-component systems are conserved among many γ-Proteobacteria. They modulate diverse biological activities relating to metabolism, motility, and protease activity, by which they eventually influence the extent of virulence, in the case of pathogens [18]. This system positively controls the expression of small RNAs, which then bind to the RNA binding protein CsrA, in order to modulate translation of the target genes. In a previous study, the *ΔvarA* mutant *V. vulnificus* revealed a lower abundance of these small RNAs [25]. The *ΔvarA* mutant constructed in this study also showed lower transcript levels of the small RNAs, *csrB1*, *csrB2*, *csrB3*, and *csrC* by Northern blot analysis and fusion assays, as expected (data not shown).

**Figure 1:**
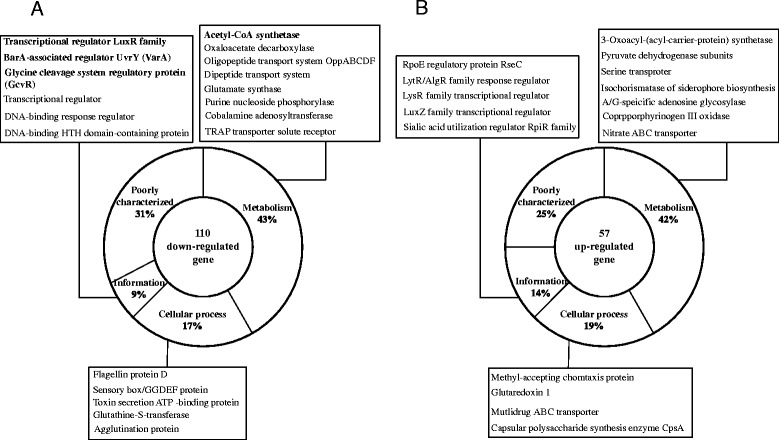
*V. vulnificus* genes showing altered expression in the Δ*varA* mutant as determined by comparative transcriptome analysis with wild type. **A -** A diagram of the down-regulated *V. vulnificus* genes in the Δ*varA* mutant. When the normalized expression relative to wild type was confined to be <0.5 with a statistical significance [P-value <0.05 by one-sample Student *t*-test using MultiExperiment Viewer (The Institute for Genome Research, http://www.tm4.org/mev.html) 4.8.1 version], 110 gene were selected and then divided into four functional categories, i.e., metabolism, cellular process, information process, and poorly characterized genes. For each category, representative genes are presented along with the percentage; **B** - A diagram of the up-regulated *V. vulnificus* gene in the Δ*varA* mutant. When the normalized expression relative to wild type was confined to be <2 with a statistical significance [P-value <0.05 by one-sample Student *t*-test using MultiExperiment Viewer (The Institute for Genome Research, http://www.tm4.org/mev.html) 4.8.1 version], 57 genes were selected and then divided into four functional categories, i.e., metabolism, cellular process, information process, and poorly characterized genes. For each category, representative genes are presented along with the percentage.

Microarray assays on transcriptomes of the *ΔvarA* mutant and wild-type *V. vulnificus* revealed 167 genes showing altered expression in the mutant (110 and 57 as down-regulated and up-regulated genes, respectively) when the normalized expression relative to wild type was confined to be <0.5 or >2 with a statistical significance (P-value <0.05) (Figure 1 and Additional file 1: Table S1). As expected, the transcript level of the *varA* gene in the *ΔvarA* mutant was not detected. Both down-regulated and up-regulated genes in the *ΔvarA* mutant were evaluated by Cluster of Orthologous Groups (COG) designation [28], and grouped into four functional categories, i.e., metabolism, cellular process, information process, and poorly characterized genes.

The largest group of both down- and up-regulated genes belonged to metabolism (42-43%), which covers various metabolic pathways for energy, carbon, nucleotide, lipid, amino acid, cofactor and secondary metabolites. One of the down-regulated genes was found to encode acetyl-CoA synthetase (Figure 1A). Interestingly, seven components involved in oligopeptide transport system (OppABCDF) and three subunits of the dipeptide transporter (Dpp) were concomitantly identified as down-regulated proteins in the *ΔvarA* mutant. A significant portion of the genes showing altered expression in the *ΔvarA* mutant (25-31%) encoded hypothetical proteins or putative proteins with biochemical activities. Another group of genes showing lower or higher expression in the *ΔvarA* mutant encodes proteins involved in cellular processes such as motility, signal transduction, resistance to oxidative stress and toxin secretion. Comparative transcriptome analysis also showed that several transcription factors were differentially expressed in the *ΔvarA* mutant compared to the wild type. Down-regulated genes in the *ΔvarA* mutant encode putative transcriptional regulators with conserved domains (annotated as a transcriptional regulator, a DNA-binding response regulator, a DNA-binding HTH domain-containing protein, and a LuxR family transcriptional regulator). One of the down-regulated genes encodes a negative regulator GcvR for the glycine cleavage system, a well-known metabolic pathway involved in glycine degradation [29]. Up-regulated proteins in the *ΔvarA* mutant include RseC, a regulator of the extracytoplasmic stress response sigma factor, sigmaE [30,31]. Transcripts of two putative transcriptional factors containing the domains conserved in LytR/AlgR and LysR family proteins were found at a higher level in the *ΔvarA* mutant. Another up-regulated gene in the *ΔvarA* mutant encodes the LuxZ homologous protein involved in bioluminescence of *Photobacterium* [32]. Lastly, higher expression of the *rpiR* gene was detected in the *ΔvarA* mutant, which encodes a regulatory protein with the binding domain for phosphosugar [33].

In this study, a putative LuxR-type transcription factor (VVMO6_00196) showing decreased expression in the *ΔvarA* mutant was chosen for further investigation. The transcript level of this LuxR-type regulator was measured in both wild type and *ΔvarA* mutant by quantitative real-time PCR. As expected from the microarray data, a relative transcript level of this gene in the *ΔvarA* mutant was 42 ± 9% of the wild-type level (Student *t-*test, P-value = 0.0008).

### Identification of target gene(s) controlled by the putative LuxR-type transcription factor

**Figure 2:**
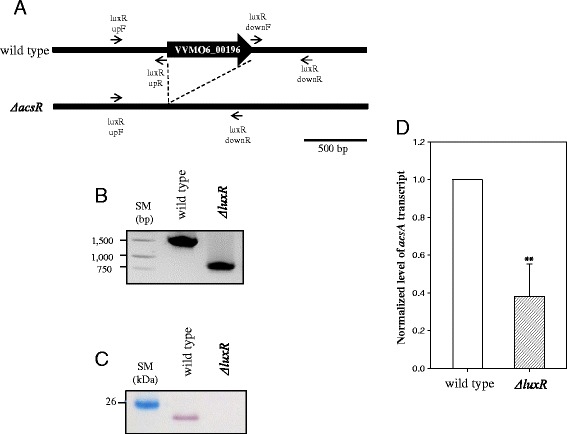
Identification of *acsA* as a down-expressed gene in mutant devoid of the LuxR-type regulator. **A** - Construction of *V. vulnificus* mutant defective in the LuxR-type transcription factor by using two sets of primers (indicated by horizontal arrows with the primer names listed in Additional file 4: Table S2) to delete VVMO6_00196. A bar represents the length of DNA equivalent to 500 bp; **B** - Deletion of the corresponding gene was examined by PCR using a pair of primers, luxRupF and luxRdownR; **C** - Confirmation of the deletion mutant. No production of the LuxR-type transcription factor in the mutant was confirmed by western blot analysis using polyclonal antibodies raised against recombinant protein of the LuxR-type transcription factor; **D** - Real-time PCR assay measuring the relative transcript level of *acsA* in mutant defective in the LuxR-type transcription factor. Relative transcript levels of *acsA* in wild-type and mutant strains were estimated as described in Figure 1. Data are presented as the mean ± standard deviation from three independent experiments. Statistical analyses were performed using Student *t*-test to evaluate the statistical significance of the results. A datum with P <0.01 is indicated with two asterisks. SM indicates DNA or protein size markers.

In a subsequent experiment, we constructed a mutant *V. vulnificus* devoid of the LuxR-type regulator, the deletion of which was confirmed by PCR using specific primers annealed to upstream and downstream regions of this gene (Figure 2A and B). This mutant was also examined by western blot using polyclonal antibodies against the recombinant protein of the LuxR-type regulator (Figure 2C). As expected, the mutant did not show any immunoreactive band around 23 kDa, which was present in the extract of the wild type.

Comparative transcriptome analysis of this mutant was performed using a *V. vulnificus* DNA microarray (Table 1). As expected, the level of the *luxR* transcript was too low to be detected in the *ΔluxR* mutant transcriptome. Beside the *luxR* gene, twenty-three genes demonstrated altered expression in the *ΔluxR* mutant with statistical significances (11 down- and 12 up-regulated genes). Three genes showing decreased expression in the mutant encode metabolic enzymes such as acetyl-CoA synthetase, phosphoenolpyruvate carboxylase, and aspartate carbamoyltransferase. One of the down-regulated genes encodes the MarC protein, which had been thought as a multiple antibiotic resistance protein [34], but it was later found to be unrelated with the antibiotic resistance [35]. It is most notable that the *msh* transcripts encoding five components of the mannose-sensitive hemagglutinin (MASH) pilus, were found at a lower level in the *ΔluxR* mutant. Another down-regulated gene encodes a homologous protein to *E. coli* DEAD-box protein A, an RNA helicase involved in structural rearrangement of ribosomal RNA [36].

**Table 1:** **Genes showing altered expression in the** *Δ* ***luxR*** **mutant compared to wild-type** ***V. vulnificus***

**Identification**	**ORF description**	**Relative expression** ^**a**^ **(P-value)**	**COG** ^**b**^
VVMO6_00196	Transcriptional regulator LuxR family	0.007(0.000)	K
VVMO6_00187	Acetyl-coenzyme A synthetase	0.51 (0.037)	I
VVMO6_00310	Phosphoenolpyruvate caroxylase	0.54 (0.032)	C
VVMO6_00392	Aspartate carbamoyltransferase	0.53 (0.027)	E
VVMO6_00195	Multiple antibiotic resistance protein MarC	0.08 (0.049)	V
VVMO6_00366	MASHA biogenesis protein MshM	0.25 (0.007)	U
VVMO6_00368	MASHA biogenesis protein MshE	0.25 (0.001)	U
VVMO6_00369	MASHA biogenesis protein MshG	0.30 (0.009)	U
VVMO6_00370	MASHA biogenesis protein MshF	0.32 (0.008)	U
VVMO6_00372	MASHA pilin protein MshA	0.50 (0.045)	U
VVMO6_03644	Cold-shock DEAD-box protein A	0.54 (0.031)	J
VVMO6_01193	Head-to-tail joining protein	0.53 (0.010)	R
VVMO6_01633	Glycosyltransferase SypQ	2.74 (0.040)	G
VVMO6_03716	Inosine-guanosine kinase	2.35 (0.041)	F
VVMO6_04198	Non-ribosomal peptide synthetase modules siderophore biosynthesis	3.80 (0.032)	Q
VVMO6_01224	Redox protein	2.09 (0.036)	V
VVMO6_03898	VgrG protein	2.30 (0.020)	U
VVMO6_04555	Positive regulator of CheA protein (CheW)	2.16 (0.018)	N
VVMO6_03446	MutT/nudix family protein	2.02 (0.025)	L
VVMO6_01214	Hypothetical protein	2.14 (0.043)	S
VVMO6_02404	Hypothetical protein	2.57 (0.008)	S
VVMO6_03566	Hypothetical protein	2.46 (0.043)	S

^a^Normalized transcript level in the *ΔacsR* mutant to the wild type as determined by microarray analysis. The presented data include the normalized values <0.55 or >2 with P <0.05.

^b^COGs: J, translation, ribosomal structure and biogenesis; L, replication, recombination and repair; V. defense mechanisms; N, cell motility; U, intracellular trafficking, secretion and vesicular transport; C, energy production and conversion; G, carbohydrate transport and metabolism; E, amino acid transport and metabolism; F, nucleotide transport and metabolism; I, lipid transport and metabolism; Q, secondary metabolite biosynthesis, transport and catabolism; R, general function prediction only; S, function unknown.

Up-regulated genes in the *ΔluxR* mutant also encode three metabolic enzymes; a glycosyltransferase SypQ for poly-N-acetylglucosamine biosynthesis [37], an inosine-guanosine kinase for nucleotide metabolism, and an enzyme for siderophore biosynthesis. Another up-regulated gene in the *ΔluxR* mutant encodes a protein homologous to VgrG protein, a component comprising the type VI secretion system in gram-negative bacteria [38]. Interestingly, the gene encoding CheW homologous protein was transcribed more in the *ΔluxR* mutant. CheW functions as a cytoplasmic adaptor protein to form the bacterial chemosensory array along with CheA protein [39]. In addition, three genes encoding hypothetical proteins (VVMO6_01214, VVMO6_02404, and VVMO6_03566) showed increased expression in the *ΔluxR* mutant.

One of the down-regulated proteins in this mutant was acetyl-CoA synthetase [acetate:CoA ligase (AMP-forming) EC 6.2.1.1], which catalyzes a conversion of acetate to acetyl-CoA. The database of the *V. vulnificus* MO6-24/O genome showed that an ORF (VVMO6_00187) encoding acetyl-CoA synthetase is *acsA* gene. Down-expression of the *acsA* gene in the mutant defective in the LuxR-type regulator was confirmed by real-time PCR (Figure 2D). The *acsA* transcript level in this mutant was 38 ± 17% of the wild type, indicating that this LuxR-type protein is a positive regulator for expression of acetyl-CoA synthetase. Therefore, we named the ORF encoding this LuxR-type regulator *acsR*, a regulator of the *acsA* gene expression.

### Regulation of *acsA* expression by AcsR

**Figure 3:**
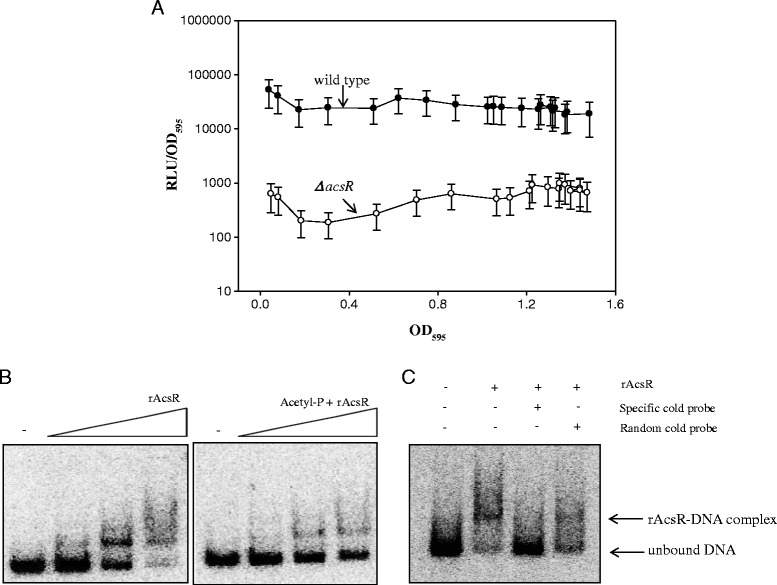
Role of AcsR in *acsA* gene expression. **A** - Expression of a transcription reporter fusion of *acsA*. Wild-type and *ΔacsR* strains carrying an *acsA::luxAB* transcription fusion were grown in AB medium supplemented with 3 μg/ml tetracycline, and luminescence produced by each strain was determined using a luminometer during the whole growth period. The specific luciferase activities were presented by plotting the normalized values as relative light units (RLU) per biomass (OD_595_); **B** - Gel-shift assay for binding of rAcsR to the regulatory region of *acsA*. A labeled 284-bp DNA fragment including an upstream region of *acsA* was incubated with various concentrations of rAcsR (0.5 ~ 5 μM). The reaction mixtures were separated on a 6% non-denaturing polyacrylamide gel. Phosphorylated rAcsR was used in another set of gel-shift assays. rAcsR (60 μg/ml) was incubated with for 1 h at 30°C in a buffer containing 100 mM Tris-HCl (pH 7.0), 10 mM MgCl_2_, 125 mM KCl, and 50 mM dilithium acetyl-phosphate (Sigma); **C** – Competition assay for AcsR-*acsA* promoter DNA. For competition analysis, the identical but unlabeled DNA fragment (competitor DNA) was included in the reactions with 1 μM rAcsR at concentrations at 760 nM. As a nonspecific control, the *gap* DNA was added into the binding reaction at 760 nM.

The effect of the *acsR* mutation on *acsA* gene expression was monitored using an *acsA::luxAB* transcriptional reporter fusion during the entire growth cycle of *V. vulnificus* (Figure 3A). The *ΔacsR* mutant strain carrying pHKacsA::luxAB showed basal levels of luciferase activity which were 50 ~ 100-folds lower than the wild-type strain carrying the same reporter, indicating that *acsA* gene expression is activated by AcsR.

To determine whether the effect of AcsR on *acsA* expression is mediated by direct binding to the regulatory region of the *acsA* gene, a gel-shift assay was performed using recombinant AcsR protein (rAcsR) and a 284-bp DNA fragment that included an upstream region of the *acsA* gene (Figure 3B, left panel). Addition of rAcsR resulted in retarded mobility of the DNA fragment due to the complex formation of rAcsR and probe DNA in an AcsR dose-dependent manner. Since AcsR is a putative response regulator of two-component signal transduction system, the phosphorylated form of rAcsR was prepared by pre-incubation with acetyl-phosphate, and then used for a gel-shift assay (Figure 3B, right panel). No apparent increase was observed in the binding to the *acsA* promoter DNA. Rather that, the degree of DNA binding seemed to be reduced in case of rAcsR treated with acetyl-phosphate. Thus, rAcsR was used for the subsequent gel-shift assays without acetyl-phosphate treatment. Binding of rAcsR to the DNA was found to be specific, because excess unlabeled probe DNA abolished the retarded bands (Figure 3C). On the other hand, inclusion of unlabeled *gap* DNA did not disrupt complex formation between rAcsR and the *acsA* promoter.

### Role of AcsS, a putative sensor kinase in expression of the *acsA* gene

**Figure 4:**
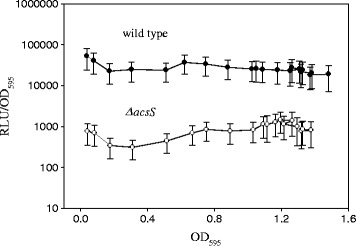
Role of AcsS in *acsA* gene expression. Expression of a transcription reporter fusion of *acsA*. Light production was determined from the *ΔacsS* strain carrying an *acsA::luxAB* transcription fusion. The specific luciferase activities were presented by plotting the normalized values as relative light units (RLU) per biomass (OD_595_).

It has been proposed that in *Shewanella oneidensis*, a regulatory system composed of SO_2742 (sensor kinase) and SO_2648 (response regulator) controls acetate metabolism by positively regulating the expression of SO_2743 (acetyl-CoA synthetase) [40]. Amino acid sequences of SO_2648 shows 56% identity to those of *V. vulnificus* AcsR (VVMO6_00196). In addition, we found that there is an ORF (VVMO6_00191) showing 46% identity with the amino acid sequences of the cognate sensor kinase, SO_2742. Therefore, we examined whether *acsA* expression of *V. vulnificus* is also regulated by this putative sensor kinase by constructing a *ΔacsS* mutant (Additional file 2: Figure S1A). Deletion of the *acsS* gene in the mutant *V. vulnificus* was confirmed by PCR analysis using a set of primers specific to upstream and downstream regions of the *acsS* gene, which showed different sizes of PCR products from the mutant and wild-type strains (Additional file 2: Figure S1B). Effect of the *acsS* mutation on *acsA* gene expression was examined using the *acsA::luxAB* transcription reporter fusion (Figure 4). The *ΔacsS* mutant carrying pHKacsA::luxAB showed significantly reduced luciferase activity similar to the *ΔacsR* mutant carrying the same reporter plasmid. Thus, it appears that AcsS also controls expression of the *acsA* gene.

### Phylogenetic analysis of AcsR and AcsS proteins

**Figure 5:**
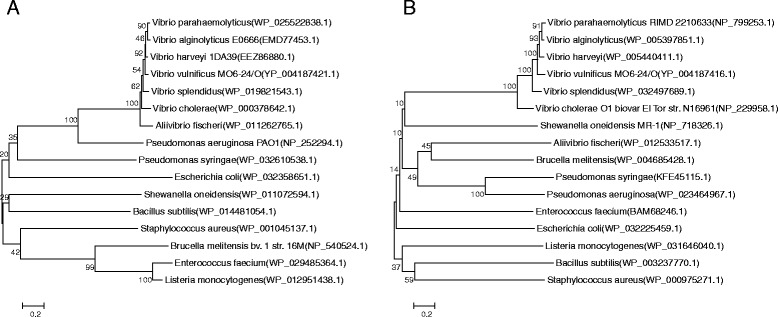
Phylogenetic relationships of the *V. vulnificus* AcsR **(A)** and AcsS **(B)** proteins. The pairwise phylogenetic distances were calculated using the Poisson correction method, and the tree was constructed using the Neighbor-Joining algorithm implemented in MEGA6 (software). The numbers at the nodes indicate the bootstrap score as percentage and are shown for frequencies at or above the threshold of 50%. The scale bar represents the expected number of substitutions per amino acid position. Sequences obtained from GenBank included *Vibrio vulnificus* (YP_004187421.1), *V. parahaemolyticus* (WP_025522838.1), *V. cholera* (WP_000378642.1), *V. alginolyticus* (EMD77453.1), *V. harveyi* (EEZ86880.1), *V. splendidus* (WP_019821543.1), *Aliivibrio fischeri* (WP_011262765.1), *Shewanella oneidensis* (WP_011072594.1), *Pseudomonas syringae* (WP_032610538.1), *P. aeruginosa* (NP_252294.1), *Brucella melitensis* (NP_540524.1), *Escherichia coli* (WP_032358651.1), *Bacillus subtilis* (WP_014481054.1), *Staphylococcus aureus* (WP_001045137.1), *Enterococcus faecium* (WP_029485364.1), *Listeria monocytogenes* (WP_012951438.1) for AcsR analysis. Information on the AcsS proteins included *V. vulnificus* (YP_004187416.1), *V. parahaemolyticus* (NP_799253.1), *V. cholera* (NP_229958.1), *V. alginolyticus* (WP_005397851.1), *V. harveyi* (WP_005440411.1), *V. splendidus* (WP_032497689.1), *A. fischeri* (WP_012533517.1), *S. oneidensis* (NP_718326.1), *P. syringae* (KFE45115.1), *P. aeruginosa* (WP_023464967.1), *B. melitensis* (WP_004685428.1), *E. coli* (WP_032225459.1), *B. subtilis* (WP_003237770.1), *S. aureus* (WP_000975271.1), *E. faecium* (BAM68246.1), *L. monocytogenes* (WP_031646040.1).

Phylogeny reconstitution using Neighbor-Joining analysis revealed a cluster containing AcsR proteins of various *Vibrio* spp., including *V. parahaemolyticus*, *V. alginolyticus*, *V. harveyi*, *V. vulnificus*, *V. splendidus*, *V. cholerae*, and *Vibrio fischeri* (also known as *Aliivibrio fischeri*) (Figure 5A). Other AcsR proteins derived from *Pseudomonas* spp. and *E. coli* showed closer relationship with these *Vibrio* AcsR proteins than those of Gram-positive bacteria. In the same manner, AcsS proteins of *Vibrio* spp. also form a clade (Figure 5B); however, AcsS proteins of *Pseudomonas* spp. and *E. coli* are grouped with those derived from *Brucella melitensis* and Gram-positive bacteria, respectively.

### Role of AcsA, AcsR, and AcsS in bacterial growth in acetate-minimal medium

**Figure 6:**
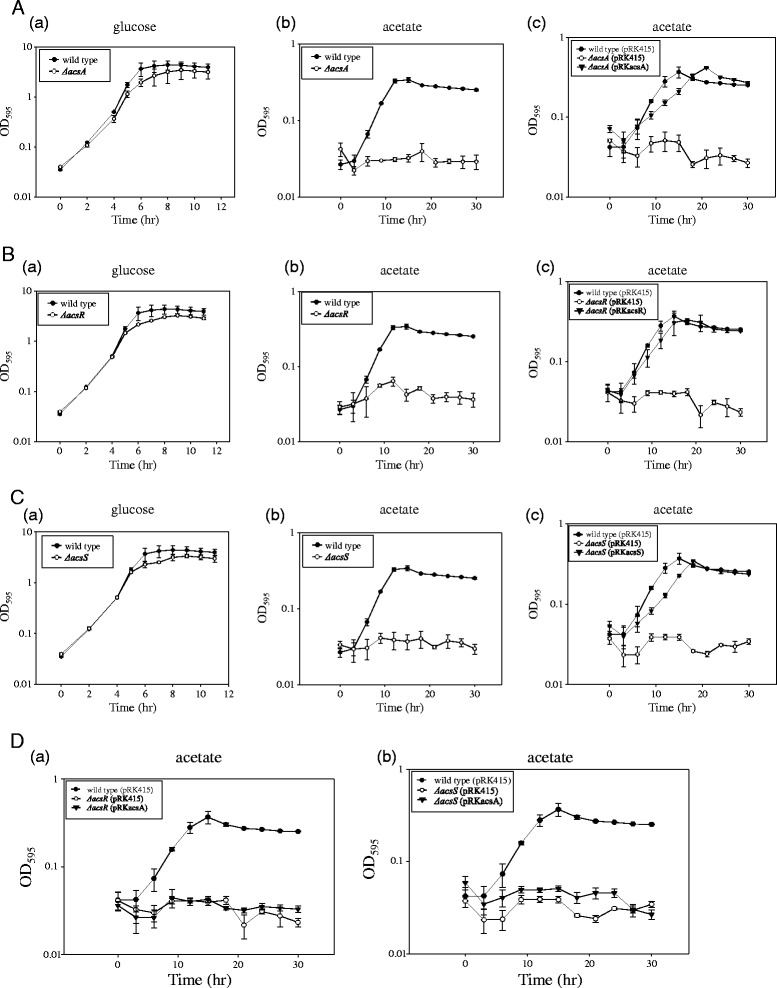
Role of AcsA, AcsS, and AcsR in *V. vulnificus* growth using glucose or acetate as a carbon source*.* **A** - Role of *acsA* in bacterial growth in the presence of glucose or acetate as a carbon source. Wild-type and *ΔacsA* strains were inoculated in the minimal medium containing glucose (a) or acetate (b), and then bacterial growth was monitored using a spectrophotometer (OD_595_). To confirm the growth defect of the mutant was caused by a deletion of the *acsA* gene, the *ΔacsA* mutant supplied with a broad-host-range vector containing the original *acsA* gene (pRKacsA) was grown in an acetate-minimal medium supplemented with 3 μg/ml tetracycline (c). For comparison, wild type and *ΔacsA* carrying the vector plasmid (pRK415) were included in this assay; **B** – Growth of wild-type and *ΔacsR* strains; **C** – Growth of wild-type and *ΔacsS* strains; *V. vulnificus* strains were inoculated to the minimal medium containing glucose (a) or acetate (b), and then bacterial growth was monitored using a spectrophotometer (OD_595_). To confirm the growth defect of each mutant was caused by a deletion of the *acsR* or *acsS* gene, each mutant supplied with a broad-host-range vector containing the original *acsR* or *acsS* genes (pRKacsR or pRKacsS, respectively) was grown in acetate-minimal medium supplemented with 3 μg/ml tetracycline (c). For comparison, wild type and mutants carrying the vector plasmid (pRK415) were also included in this assay; **D** – Complementation of *ΔacsR* (a) and *ΔacsS* (b) mutant *V. vulnificus* with a broad-host-range vector containing the original *acsA* gene (pRKacsA) and their growth in acetate minimal medium.

A *V. vulnificus* strain lacking acetyl-CoA synthetase (VVMO6_00187) was constructed by deleting the *acsA* gene (Additional file 3: Figure S2A). Successful deletion of the internal region of the *acsA* gene in the chromosome of *V. vulnificus* was confirmed by PCR showing a smaller PCR product from the *ΔacsA* mutant than that from the wild type (Additional file 3: Figure S2B). To assess the physiological role of AcsA, growth of the *ΔacsA* mutant was compared with wild type in a medium containing glucose or acetate as the sole carbon source [Figure 6A, (a) and (b)]. While the *ΔacsA* mutant retained the ability to grow in glucose-minimal medium at ~80% of the wild type, it did not show any apparent ability to use acetate for its growth. Growth of the *ΔacsA* mutant in acetate-minimal medium returned to that of the wild type when the *ΔacsA* mutant strain was complemented by carriage of a copy of the original *acsA* gene [Figure 6A, (c)].

Mutant *V. vulnificus* strains devoid of either *acsR* [response regulator; Figure 6B, (a) and (b)] or *acsS* [sensor histidine kinase; Figure 6C, (a) and (b)] showed defective growth in acetate-minimal medium compared to wild-type *V. vulnificus*. When the *ΔacsR* mutant strain was complemented with the intact *acsR* gene, the mutant gained the ability to use acetate as a carbon source [Figure 6B, (c)]. In the same manner, a complemented *ΔacsS* mutant also exhibited the ability to grow in acetate-minimal medium at almost the same level as the wild-type growth [Figure 6C, (c)].

These results suggest that growth defect of *ΔacsR* and *ΔacsS* mutant in acetate-minimal medium is caused by attenuated production of acetyl-CoA synthetase. To examine this possibility, *ΔacsR* and *ΔacsS* mutant strains carrying a copy of the original *acsA* gene were constructed, and monitored for their growth in acetate-minimal medium (Figure 6D). However, growth of both the *ΔacsR* and *ΔacsS* mutant in acetate-minimal medium was not restored when these strains had the plasmid containing the *acsA* gene suggesting that control of acetate metabolism by AcsS/AcsR extends beyond regulation of *acsA* expression.

### cAMP-independent catabolite repression of *acsA* expression

**Figure 7:**
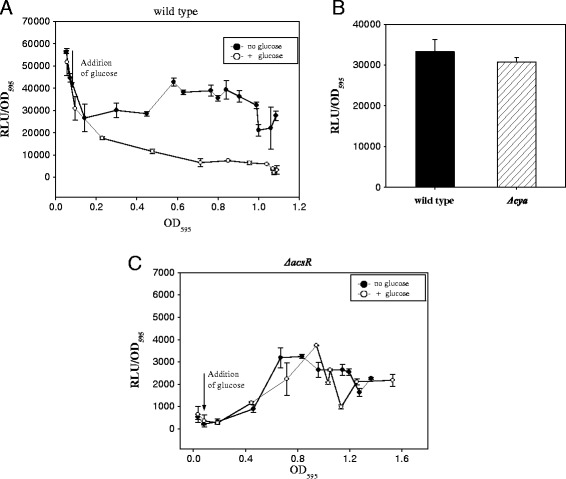
Glucose effect on expression of a transcription reporter fusion of *acsA*. **A** - Wild type carrying an *acsA::luxAB* transcription fusion was inoculated in AB medium supplemented with 3 μg/ml tetracycline and 22 mM glucose was added at the indicated time point (vertical arrows); **B** -Effect of a *cya* mutation on expression of a transcription reporter fusion of *acsA*. Wild type and *Δcya* mutant carrying an *acsA::luxAB* transcription fusion were inoculated in AB medium supplemented with 3 μg/ml tetracycline and grown up to OD_595_ = 1.0; **C** - *ΔacsR* mutant carrying an *acsA::luxAB* transcription fusion were inoculated in AB medium supplemented with 3 μg/ml tetracycline and 22 mM glucose was added at the indicated time point (vertical arrows). Luminescence produced by each strain harvested at the exponential phase was expressed by specific luciferase activities [relative light units (RLU) per biomass (OD_595_)] as described in Figure 3.

Acetyl-CoA synthetase is required for normal levels of *V. vulnificus* growth in media with acetate as the sole source, which was evidenced by the defective growth of the *ΔacsA* mutant in an acetate medium (Figure 6A). This mutant, however, did not show any defect in growth in a glucose-minimal medium. Thus, *acsA* expression may not be induced when cells are growing in the presence of other carbon sources such as glucose. This speculation implies the presence of another regulatory pathway for *acsA* expression in *V. vulnificus*. Therefore, expression of the *acsA* gene was monitored in wild type growing in a glycerol-minimal medium (Figure 7A). Addition of glucose to the glycerol-minimal medium reduced the expression of the *acsA::luxAB* fusion, indicating that *acsA* expression might be under the regulation of catabolite repression. In a subsequent experiment, we examined whether catabolite repression of *acsA::luxAB* activity is mediated by a well-known regulator, cAMP-CRP. The reporter plasmid of the *acsA::luxAB* fusion was transferred into a *Δcya* mutant, which was unable to synthesize cyclic AMP [41]. There was no difference in *acsA::luxAB* expression in *Δcya* mutant and the wild type during the entire growth cycle of *V. vulnificus* including the phase at OD_595_ = 1.0 shown in Figure 7B. This result may indicate that repression of *acsA* expression by glucose is not mediated by cAMP-CRP in *V. vulnificus*. To investigate the mechanism underlying catabolite repression-like regulation of *acsA* expression, glucose was added to *ΔacsR* mutant growing with glycerol as a carbon source. Luciferase activities of *acsA::luxAB* were basal during the entire growth of the *ΔacsR* mutant in glycerol-minimal medium. Addition of glucose, however, did not cause any repressive effect on *acsA* expression (Figure 7C), which implies that the glucose effect on *acsA* expression might be mediated by AcsR.

## Discussion

The ability of a bacterium to use a specific carbon source is tightly controlled to assure the most efficient use of metabolic pathways under specific conditions, including those of characteristic of the host environment. Acetyl-CoA synthetase is an enzyme that converts acetate into acetyl-CoA, which is crucial in utilizing acetate as a carbon source [42]. *E. coli* is able to grow by utilizing a wide range of acetate concentrations (2.5 to 50 mM), but an *acsA*-mutated *E. coli* grows poorly in media containing a relatively low concentration of acetate (<10 mM). In contrast, mutants deficient in the *ack* and *pta* genes encoding the second acetyl-CoA producing system grow poorly in a high concentration of acetate (>25 mM) [43]. A metabolic phenomenon called “acetate switch” is a good example of how microorganisms such as *E. coli* modulate their metabolism under various growth conditions [44]. During exponential growth, the bacteria consume carbon sources such as glucose via the Ack-Pta system in order to produce and excrete acetate. When these acetogenic sugars become exhausted, the cells then begin to import and utilize environmental acetate via the action of acetyl-CoA synthetase. The role of acetyl-CoA synthetase varies according to the metabolic versatility of the microorganism. A mutant *P. aeruginosa* unable to use ethanol had lost its acetyl-CoA synthetase activity [45]. A study using an *acsA::lacZ* fusion indicated that transcription of the *acsA* gene is induced by acetate in an ErdR-dependent manner [46]. Interestingly, the amino acid sequence of ErdR shows a 52% identity with that of AcsR of *V. vulnificus*. In the case of *V. fischeri*, a symbiotic microbe with an squid, the *ainS* mutant defective in production of octanoyl-homoserine lactone also lost the ability to perform an “acetate switch” because it had defects in the expression of the *acsA* gene [47]. Their study demonstrated that acetate switch is controlled by quorum sensing and plays a role in light organ symbiosis by *V. fischeri*.

Experimental information regarding the metabolic versatility of *V. vulnificus* has not yet been available in the metabolic pathways utilizing acetate*.* When acetate was provided as a sole carbon source to *V. vulnificus*, the *acsA* gene product was essential for its growth (Figure 6A). AcsA is also used by the cell to recover catabolically produced acetate excreted during sugar metabolism. When the concentration of acetate was high (>20 mM), however, growth of wild-type *V. vulnificus* was affected (Kim, M-J. and Park, S-J., unpublished data). Therefore, *V. vulnificus* mutants devoid of *ack* and/or *pta* genes need to be characterized to fully understand acetate metabolism of *V. vulnificus* by comparing it to the growth of the *ΔacsA* mutant. These *ΔacsA*, *ΔacsR*, and *ΔacsS* mutant *V. vulnificus* strains should be examined to see whether they can perform the “acetate switch”, and this process is also regulated by quorum sensing in *V. vulnifucus*.

In *E. coli*, *acsA* expression is repressed by glucose, and this catabolite repression is mediated by CRP-cAMP [48]. Although *acsA* expression was also repressed by the addition of glucose in *V. vulnificus* (Figure 7A), catabolite repression of *acsA* expression does not appear to be mediated by cAMP (Figure 7B). Interestingly, cAMP-independent catabolite repression of *acsA* expression was not observed in the *acsR* mutant (Figure 7C), which already had a greatly reduced expression. Thus, this study cannot rule out the possibility that AcsR might be involved in catabolite repression-like regulation of *acsA* expression. The regulatory mechanism underlying this catabolite repression needs to be elucidated in future studies. Transcription factors mediating catabolite repression via cAMP-CRP-independent manners have been reported in some microorganisms. AccR is known as a master regulator involved in carbon catabolite repression of the anaerobic catabolism of aromatic compounds in *Azorcus* sp. [49]. In *Pseudomonas,* Crc, a translational repressor of multiple pathways linked to catabolite repression is known to be modulated by small RNAs, *crcZ* and *crcY* [50].

Expression of the acetyl-CoA synthetase gene is significantly reduced in *V. vulnificus* devoid of *acsR* or *acsS* using the *luxAB*-transcriptional reporter fused with the regulatory region of *acsA* (Figures 3A and 4). While the *acsA* mRNA level in the *acsR* mutant was decreased to 38% of wild type (Figure 2D), luciferase activity of the *acsA::luxAB* fusion was dramatically reduced in the mutant (Figure 3A). This discrepancy may be derived from the drawback of the *acsA::luxAB* fusion plasmid. Because this fusion plasmid is present in multiple copies, its expression level could be amplified or variable under certain conditions. Alternatively, in addition to direct transcriptional regulation, AcsS/AcsR might indirectly affect *acsA* expression at the post-transcriptional level.

Absence of the AcsR or AcsS protein resulted in a severe growth defect in the presence of acetate as a carbon source (Figure 6B and C). The subsequent experiment did not provide the evidence that the growth defect of the *ΔacsR* and *ΔacsS* mutants was caused from bacterial inability to express acetyl-CoA synthetase (Figure 6D). It is possible that the *acsA* gene in the complementation plasmid pRKacsA fails to express in the *ΔacsR* and *ΔacsS* mutants. Otherwise, Acs activity may be differentially affected in the *ΔacsR* and *ΔacsS* mutants from the wild type or *ΔacsA* mutant at a post-transcriptional level. In any cases, these data suggest that AcsR and AcsS are necessary for the *acsA* expression or Acs activity.

The positive effect of AcsR in *acsA* expression occurred through a direct interaction between this transcriptional factor and the regulatory region of *acsA* as shown in gel-shift assays (Figure 3B and C). It remains to be elucidated whether AcsR functions as a cognate response regulator of AscS in *acsA* transcription.

AcsR was found as a down-expressed protein in the *ΔvarA* mutant *V. vulnificus* along with 166 other genes showing the altered expression (Figure 1 and Additional file 1: Table S1). Both down- and up-regulated genes were found in the *ΔvarA* mutant as reported in the transcriptome profiling of the *ΔuvrY* mutant of *Photorhabdus luminescens*, a *varA* homologous gene of the insect pathogen [51]. The comparative transcriptomic analysis between wild type and *ΔuvrY* indicated that UvrY negatively regulates flagella formation/motility, and iron acquisition, and positively regulates other processes, such as protease formation, resistance against oxidative stresses, and host colonization.

Initially AcsR was identified as a down-expressed clone in the *ΔvarA* mutant (Figure 1A and Additional file 1: Table S1), and the *acsA* gene was subsequently identified as a down-regulated gene in the *ΔacsR* mutant (Table 1 and Figure 2D). Luciferase activity of the *acsA::luxAB* fusion was also significantly reduced in the *ΔvarA* mutant *V. vulnificus* (Kim, M-J. and Park, S-J., unpublished data) indicating that the regulatory cascades for *acsA* expression are composed of the VarS/VarA system as an upstream component and the AcsR as a downstream component. It is not clear if VarA is directly involved in the expression of the *acsR* gene. It might be possible that *acsR* expression is controlled via *csrB/csrC* regulators, of which expressions are tightly regulated by VarA [19]. Alternatively, VarA could directly modulate the expression of the *acsR* gene by binding to its upstream region.

In contrast to a large number of differentially expressed genes in the *ΔvarA* mutant, only two dozen genes were found at different levels between wild type and the *ΔacsR* mutant (Table 1), indicating that AcsR has a narrower spectrum of the target genes than VarA. However, it is likely that a portion of differentially expressed genes in the *ΔvarA* mutant are not directly regulated by VarS/VarA, rather that they are directly controlled via other regulatory systems such as CsrA/*csrB*/*csrC* system functioning at downstream of the VarS/VarA system.

Down-regulated genes in the *ΔacsR* mutant include the genes encoding MASH pilus. In *V. cholerae*, formation of MASH pilus was found specifically repressed *in vivo*, and thus it is considered as anti-colonization factor [52]. The *msh* genes encoding MASH pilus were transcribed as two adjacent transcripts, i.e., the secretory genes and the structural genes [53]. ToxT protein, a key regulator for *V. cholerae* virulence, represses transcription of these *msh* genes [54]. In this study, *mshMEG* genes in the secretory operon and *mshAF* in the structural operon were found at a lower level in the *ΔacsR* mutant indicating that overall expression of *msh* genes was affected in this mutant. A possibility that AcsR activates transcription of these *msh* genes via direct binding to the two *msh* promoter regions will be examined, and if it is the case, the AcsR-mediated control of MASH in *V. vulnficus* should be evaluated for its physiological implication.

## Conclusion

Transcriptome analysis of the *ΔvarA* mutant by comparison with wild-type *V. vulnificus* led us to identify a positive transcription factor, AcsR, for acetyl-CoA synthetase. Transcription of the *acsA* gene for acetyl-CoA synthetase by AcsR and AcsS is critical for bacterial growth when using acetate as a carbon source.

## Methods

### Bacterial strains and culture conditions

The bacterial strains and plasmids used in this study are listed in Table 2. *E. coli* strains used for manipulation of various plasmid DNAs were grown at 37°C in Luria-Bertani (LB) broth or on LB agar plate supplemented with the appropriate antibiotics. *V. vulnificus* strains were cultured at 30°C in LB medium supplemented with an additional 2% NaCl (LBS). Antibiotics were used at the following concentrations: ampicillin (100 μg/ml), chloramphenicol (25 μg/ml), kanamycin (50 μg/ml), and tetracycline (15 μg/ml) for *E. coli*, and ampicillin (500 μg/ml), chloramphenicol (2 μg/ml), kanamycin (100 μg/ml), and tetracycline (3 μg/ml) for *V. vulnificus*. To measure luciferase activities derived from a *luxAB*-transcription reporter fusion, the bacterial cells of *V. vulnificus* were grown in AB medium with 1% glycerol (300 mM NaCl, 50 mM MgSO_4_, 0.2% casamino acids, 1 mM L-arginine, and 10 mM potassium phosphate, pH 7.5).

**Table 2:** **Bacterial strains and plasmids used in this study**

**Strain/plasmid**	**Genotype**	**Source/Reference**
***Escherichia coli***		
DH5α	(Φ80 *lacZ ΔM15*) *recA1 endA1 gyrA96 relA1 thi-1 hsdR17*(r_K_^−^ m_K_^−^) *supE44 deoR* (*lacZYA-argF*)*U169*	Invitrogen
BL21(DE3)	*E. coli* strain B F^−^ *dcm ompT hsdS*(r_B_^−^ m_B_^−^) *galλ*(DE3)*thi-1 thr leu tonA lacY supE recA*::	Invitrogen
SM10*λpir*	Rp4-2-Tc::Mu*λpir*, *oriT* of RP4, Km^r^ *endA1 recA1 gyrA96 thi-1 hsdR17*(r_K_^−^ m_K_^−^) *relA1supE44 Δ*(*lac-proAB*)[F’*traD36proABlacI* ^q^Z*ΔM15]*	[55]
***Vibrio vulnificus***		
MO6-24/O	Clinical isolate	[56]
MJ1	MO6-24/O, *ΔvarA*	This study
MJ2	MO6-24/O, *ΔacsR*	This study
MJ3	MO6-24/O, *ΔacsS*	This study
MJ4	MO6-24/O, *ΔacsA*	This study
KJL*Δcya*	MO6-24/O, *Δcya*	This study
**Plasmids**		
pBluescript(II)SK(+)	Cloning vector; Ap^r^, *lac* promoter (*lacZ*), f1, ColE1	Stratagene
pSKvarAU	pBluescript(II)SK(+) with 501-bp upstream region of *varA*	This study
pSKvarAUD	pSKvarAU with 520-bp downstream region of *varA*	This study
pSKacsRU	pBluescript(II)SK(+) with 410-bp upstream region of *acsR*	This study
pSKacsRUD	pSKacsRU with 424-bp downstream region of *acsR*	This study
pSKacsSU	pBluescript(II)SK(+) with 710-bp upstream region of *acsS*	This study
pSKacsSUD	pSKacsSU with 528-bp downstream region of *acsS*	This study
pSKacsAU	pBluescript(II)SK(+) with 732-bp upstream region of *acsA*	This study
pSKacsAUD	pSKacsAU with 525-bp downstream region of *acsA*	This study
pDM4	Suicide vector; *ori*R6K, Cm^r^	[57]
pDMΔcya	pDM4 containing 1,272-bp DNA of internally deleted *cya* gene	[41]
pDMΔvarA	pDM4 containing 1,021-bp SalI/XbaI fragment of pSKvarAUD	This study
pDMΔacsR	pDM4 containing 834-bp ApaI/sacI fragment of pSKacsRUD	This study
pDMΔacsS	pDM4 containing 1,238-bp XhoI/XbaI fragment of pSKacsSUD	This study
pDMΔacsA	pDM4 containing 1,953-bp XhoI/XbaI fragment of pSKacsAUD	This study
pHK0011	pRK415, a promoterless *luxAB,* Tc^r^	[58]
pHKacsA::luxAB	pHK0011 vector containing 284-bp *acsA* promoter	This study
pRK415	A broad-host-range vector; *oriT* of RP4; Tc^r^	[59]
pRKacsR	pRK415 containing636-bp *V. vulnificus acsR* ORF	This study
pRKacsS	pRK415 containing3,432-bp *V. vulnificus acsS* ORF	This study
pRKacsA	pRK415 containing1,953-bp *V. vulnificus acsA* ORF	This study
pET28b(+)	Expression vector; T7 *lac* promoter, *oriF1*; Km^r^	Novagen
pETacsR	pET28b (+) vector containing 636-bp *acsR*	This study

To compare the growth pattern of *V. vulnificus* strains, each strain was grown in an NaCl-enriched M9 minimal medium (90 mM Na_2_HPO_4_, 22 mM KH_2_PO_4_, 18 mM NH_4_Cl, 2 mM MgSO_4_, 0.1 mM CaCl_2_, and 2.5% NaCl) with either 22 mM glucose or 10 mM sodium acetate as a carbon source, and bacterial growth was monitored by measuring the optical density at 595 nm (OD_595_). Overnight cultures of various *V. vulnificus* strains were prepared in LBS, washed with an NaCl-enriched M9 minimal medium without carbon source, and then used to inoculate into the fresh medium either with glucose or acetate at OD_595_ = 0.05.

### Transcriptome analysis

A customized *V. vulnificus* DNA microarray (E-biogene) was used, which contained information of all 4,562 ORFs found in the genome of *V. vulnificus* MO6-24/O. Total RNAs were extracted from *V. vulnificus* strains grown to an OD_595_ of 1.0 using the RNeasy® Mini Kit (Qiagen). The integrity of bacterial total RNAs was checked by capillary electrophoresis with an Agilent 2100 bioanalyzer (Agilent Technologies) and further purified using the RNeasy Mini kit. cDNA probes were prepared by reverse transcription of total RNA (25 μg) in the presence of aminoallyl-dUTP and 6 μg of random primers (Invitrogen). Followed by coupling of Cy3-dye (for a reference) or Cy5-dye (for a test sample) (Amersham Pharmacia), Cy3- or Cy5-labeled cDNA probes were added for hybridizationon a microarray slide. Hybridization images on the slide were obtained using a GenePix 4000A scanner (Axon Instruments). The analysis of the microarray data was performed using GenePix Pro 6.0 (Axon Instruments). Fluorescent spots and local background intensities were quantified using Agilent GeneSpring 7.3.1 software package (Agilent Technologies) to obtain gene expression ratios (mutant versus the wild type). Agilent Feature Extraction Software (version 9.3.2.1) was used for background subtraction. Signals were calculated for both Cy3 and Cy5 channels by subtracting the median of background signals from the median of spot signal of each spot. Normalization was carried out using global loess algorithm [60] using Genowiz 4.0^TM^ (Ocimum Biosolutions). The averages of the normalized ratios were calculated by dye-normalized signals of Cy3 and Cy5 channels. All samples were assayed in three different biological replicates. All measurements were performed on three technical replicates. An one-sample Student *t*-test was calculated to test whether the mean normalized ratio for the gene is statistically significant (P-value <0.05) using MultiExperiment Viewer (The Institute for Genome Research, http://www.tm4.org/mev.html) 4.8.1 version. A putative functional role of each gene was grouped by Cluster of Orthologous Groups (COG) of protein designation [28,61].

The microarray data have been deposited in the GEO database (http://www.ncbi.nlm.nih.gov/geo) under accession no. GSE67192.

### Quantitative measurement of the transcripts of a putative LuxR-type regulator and acetyl-CoA synthetase

The cellular levels of the corresponding mRNAs were evaluated by real-time PCR. Total RNA was isolated from wild-type or mutant *V. vulnificus* strains using the RNeasy® Mini Kit and treated with the RNase-free DNase I (TaKaRa). cDNA was synthesized from 4 μg of RNA using the ImProm-II^TM^ RT system (Promega) following the manufacturer’s directions. cDNA was then analyzed with the Light Cycler 480 II Real-Time PCR System (Roche Applied Science) using LightCycler 490 DNA SYBR Green I Master (Roche Applied Science). Real-time PCR was carried out in triplicate in a 96-well plate using the specific primers listed in Additional file 4: Table S2. The *gap* gene encoding NAD-dependent glyceraldehyde-3-phosphatase of *V. vulnificus* was used as an endogenous control for the reactions.

Data are presented as mean ± standard deviation from three independent experiments. Statistical analyses for pair-wise comparison were performed using Student *t*-test (SYSTAT, SigmaPlot version 11; Systat Software Inc.) to evaluate the statistical significance of the results. Differences were considered significant when P <0.05. Data with P <0.01 are indicated with two asterisks, whereas data with P-values between 0.01 and 0.05 are indicated with a single asterisk.

### Construction of deletion mutants of *V. vulnificus* and complementation of the mutant strains

#### *ΔvarA* mutant

For construction of the *ΔvarA* mutant, the upstream region of the *varA* gene was amplified from the genomic DNA of *V. vulnificus* MO6-24/O with the primers, varAupF and varAupR (Additional file 4: Table S2). The resultant 501-bp DNA fragment was then digested with SalI and PstI and ligated into pBlueScript (II) SK (+) to produce pSKvarAU. The downstream region of the *varA* gene was amplified using the primers, varAdownF and varAdownR (Additional file 4: Table S2). The resultant DNA fragment of 520-bp was treated with PstI and XbaI and ligated into pSKvarAU to yield pSKvarAUD. The 1,021-bp SalI-XbaI DNA fragment of pSKvarAUD was transferred into a suicide vector pDM4 [57], resulting in formation of pDMΔvarA. The plasmid pDMΔvarA in SM10 λ*pir* [55] was mobilized to *V. vulnificus* MO6-24/O, and the conjugants were selected by plating the conjugation mixture of *E. coli* and *V. vulnificus* on LBS plates supplemented with 2 μg/ml chloramphenicol. A colony with characteristics indicating a double homologous recombination event (resistance to 5% sucrose and sensitivity to chloramphenicol) was further confirmed by PCR using the primers, varAupF and varAdownR and then named MJ1.

#### *ΔacsR* mutant and complementation strains

For construction of the *ΔacsR* mutant, the upstream (410-bp) and downstream (424-bp) regions of the *acsR* gene were amplified using the primer set of luxRupF/luxRupR and luxRdownF/luxRdownR, respectively (Additional file 4: Table S2). The ApaI*-*SacI DNA fragment of pSKacsRUD was transferred into pDM4 to produce pDMΔacsR, which was then used to generate the *ΔacsR* mutant, as described above. For complementation of the mutant, a 951-bp DNA fragment was amplified using acsRcomF and acsRcomR (Additional file 4: Table S2), which contains a whole *acsR* ORF and a 315-bp upstream region of the *acsR* gene. This DNA fragment was then cloned into a broad-host-range vector, pRK415 [59] to produce pRKacsR. This *acsR*^*+*^-containing plasmid was mobilized to the *ΔacsR* strain via conjugation. Wild type carrying pRK415 and the *ΔacsR* strain carrying pRK415 were also prepared in the same manner to serve as controls.

#### *ΔacsS* mutant and complementation strains

A plasmid (pSKacsSUD) was made to include the upstream (710-bp) and downstream (528-bp) regions of the *acsS* gene, which had been amplified by the following primer sets, acsSupF/acsSupR and acsSdownF/acsSdownR (Additional file 4: Table S2). The XhoI-XbaI DNA fragment of the resultant plasmid was ligated into pDM4 to produce pDMΔacsS, which was used to make the *ΔacsS* mutant, as described above. For complementation of the mutant, a 3,432-bp DNA fragment was amplified using acsScomF and acsScomR (Additional file 4: Table S2). This DNA fragment was then cloned into pRK415 to produce pRKacsS that was then mobilized to the *ΔacsS* strain.

#### *ΔacsA* mutant and complementation strains

To inactivate the *acsA* gene, the primer sets of acsAupF/acsAupR and acsAdownF/acsAdownR (Additional file 4: Table S2) were utilized to produce the 732-bp upstream and the 525-bp downstream regions of the *acsA* gene, respectively. A 1,953-bp DNA fragment of pSKacsAUD was cloned to pDM4 to make pDMΔacsA, which was used to generate an *ΔacsA* mutant. To complement the original *acsA* gene into the *ΔacsA* mutant, pRKacsA was constructed by cloning the 2,241-bp *acsA* DNA fragment into the HindIII/BamHI site of pRK415 and transferred into the *ΔacsA* strain as described above.

#### *Δcya* mutant

To delete the *cya* gene in *V. vulnificus*, pDMΔcya [41] was transferred to MO6-24/O via conjugation, and a *V. vulnificus* colony with characteristics indicating a double homologous recombination event was selected and named KJLΔcya.

### Construction of a *luxAB-*transcription reporter fusion with the *acsA* promoter and measurement of its expression

The plasmid pHKacsA::luxAB was constructed by inserting a 284-bp DNA fragment including the regulatory region for *acsA* into the upstream region of the *luxAB* gene in pHK0011 [58] by utilizing restriction sites for KpnI and BamHI. pHKacsA::luxAB in *E. coli* SM10λpir was conjugated to *ΔacsR* and wild-type strains. Aliquots of overnight-grown cultures were inoculated to fresh AB broth containing tetracycline (3 μg/ml) and incubated with shaking at 30°C.

Luciferase activity in the bacterial cells carrying these fusions was measured in the presence of 0.006% (v/v) *n*-decyl aldehyde using a luminometer (TD-20/20 Luminomter, Turners Designs). Specific bioluminescence was calculated by normalizing the relative light units (RLU) with respect to cell mass (OD_595_).

### Preparation of polyclonal antibodies against recombinant AcsR and western blot analysis

A 636-bp DNA fragment encompassing the *acsR* ORF was amplified using two primers, racsRF and racsRR (Additional file 4: Table S2), and then cloned into an expression vector, pET28b (+) (Novagen). rAcsRwas overexpressed by adding isopropyl thio-β-D-galactoside at a concentration of 1 mM and purified using a TALON® affinity column (Clontech). Purified rAcsR was used to generate polyclonal antibodies by three immunizations of SPF/VAF outbred rats (200 μg AcsR per immunization) at 3-week intervals. Cellular extracts were prepared by sonicating harvested cells in TNT buffer [10 mM Tris-HCl (pH 8.0), 150 mM NaCl, and 0.05% (v/v) Tween 20]. Cell lysates were separated by SDS-PAGE and transferred to nitrocellulose membranes (Millipore). Membranes were blocked with 5% skim-milk in Tris-buffered saline with Tween 20 (TBST; 150 mM NaCl, 50 mM Tris-HCl, and 0.1% Tween 20) and then incubated overnight at 4°C with the anti-AcsR polyclonal antibodies (1:2,000 dilution). After incubation with alkaline phosphate-conjugated secondary antibodies, immunoreactive bands were visualized using nitro blue tetrazolium and 5-bromo-4-chloro-3-indolyl phosphate.

### Gel-shift assay

A 284-bp DNA fragment including the upstream region of the *acsA* gene was labeled with [γ-^32^P]ATP using T4 polynucleotide kinase. A labeled DNA probe (225 nM) was incubated with various concentrations of rAcsR (0.5 – 5 μM) for 30 min at 37°C. After the reactions were stopped, aliquots of the reaction mixtures were separated on a 6% non-denaturing polyacrylamide gel.

To prepare phosphorylated rAcsR used for gel shift assays, rAcsR (60 μg/ml) was incubated with for 1 h at 30°C in a buffer containing 100 mM Tris-HCl (pH 7.0), 10 mM MgCl_2_, 125 mM KCl, and 50 mM dilithium acetyl phosphate (Sigma) as described [62].

For competition analysis, the identical but unlabeled DNA probe was included in the reaction mixture at a concentration of 716 nM. As a nonspecific control, the *gap* DNA encoding glyceraldehyde 3 phosphate dehydrogenase was included in the binding reaction at 716 nM.

### Phylogenetic analysis of AcsR and AcsS proteins

The evolutionary history was inferred using the Neighbor-Joining method [63]. The percentages of replicate trees in which the associated taxa clustered together in the bootstrap test (1000 replicates) were shown next to the branches [64]. The tree is drawn to scale, with branch lengths in the same units as those of the evolutionary distances used to infer the phylogenetic tree. The evolutionary distances, computed using the Poisson correction method [65], were in the units of the number of amino acid substitutions per site. Evolutionary analyses were conducted in MEGA6 [66]. The scale bar indicates the number of amino acid substitutions per site.

## Additional files

Additional file 1: Table S1.
**Genes showing altered expression in the** ***ΔvarA*** **mutant compared to wild-type** ***V. vulnificus***. Additional file 2: Figure S1.
**Construction of** ***ΔacsS*** **mutant** ***V. vulnificus.*** A - Construction of *V. vulnificus* mutant defective in *acsS* by using two sets of primers (indicated by horizontal arrows with the primer names listed in Additional file 4: Table S2) to delete VVMO6_00191. A bar represents the length of DNA equivalent to 500 bp; B - Deletion of the corresponding gene was examined by PCR using a pair of primers, acsSupF and acsSdownR. SM indicates DNA size markers. Additional file 3: Figure S2.
**Construction of** ***ΔacsA*** **mutant** ***V. vulnificus.*** A - Construction of *V. vulnificus* mutant defective in *acsA* by using two sets of primers (indicated by horizontal arrows with the primer names listed in Additional file 4: Table S2) to delete VVMO6_00187. A bar represents the length of DNA equivalent to 500 bp; B - Deletion of the corresponding gene was examined by PCR using a pair of primers, acsAupF and acsAdownR. SM indicates DNA size markers. Additional file 4: Table S2.
**Oligonucleotide primers used in this study.**

## Abbreviations

Acs: Acetyl-CoA synthetase
Ack: Acetate kinase
Pta: Phosphtransacetylase
rAcsR: Recombinant AcsR
LB: Luria-Bertani
RLU: Relative light unit
COG: CLUSTER of orthologous groups
MASH: Mannose-sensitive hemagglutinin
