# Supplementary material for: Role of AcsR in expression of the acetyl-CoA synthetase gene in Vibrio vulnificus
Source: BMC Microbiol. 2015 Apr 12;15:86. doi: 10.1186/s12866-015-0418-4 (PMC4409781; doi:10.1186/s12866-015-0418-4)
Supplement: Additional file 1: Table S1. — Genes showing altered expression in the ΔvarA mutant compared to wild-type V. vulnificus. [file 12866_2015_418_MOESM1_ESM.docx]

**Supplementary Table 1 Genes showing altered expression in the** *Δ****varA* mutant compared to wild-type *V. vulnificus***

| Identification | ORF description | Relative expression^a^  (P-value) | COG^b^ |
| --- | --- | --- | --- |
| **Metabolism** | | | |
| VVMO6_00187 | Acetyl-coenzyme A synthetase | 0.50 (0.007) | I |
| VVMO6_00260 | Succinylglutamic semialdehyde dehydrogenase | 0.50 (0.005) | C |
| VVMO6_00263 | Phosphoribulokinase-like protein | 0.50 (0.002) | C |
| VVMO6_00269 | Glutathione-regulated potassium-efflux system protein KefB | 0.49 (0.001) | P |
| VVMO6_00506 | Oxaloacetate decarboxylase α-subunit | 0.37 (0.006) | C |
| VVMO6_00507 | Oxaloacetate decarboxylase β-subunit | 0.28 (0.035) | C |
| VVMO6_00562 | Chitobiose phosphorylase | 0.41 (0.003) | G |
| VVMO6_00624 | Phosphopentomutase | 0.41 (0.046) | G |
| VVMO6_01122 | Aspartate aminotransferase | 0.48 (0.008) | E |
| VVMO6_01269 | Histidine ammonia-lyase | 0.47 (0.017) | E |
| VVMO6_01271 | Formiminoglutamase | 0.37 (0.025) | E |
| VVMO6_01492 | Iron-sulfur cluster-binding protein | 0.41 (0.019) | C |
| VVMO6_01493 | Formate dehydrogenase-specific chaperone | 0.40 (0.030) | C |
| VVMO6_01663 | Glutamate aspartate periplasmic binding protein GltI | 0.42 (0.027) | E |
| VVMO6_01715 | Amino acid transporter | 0.42 (0.007) | E |
| VVMO6_01721 | Fumarylacetoacetase | 0.48 (0.025) | Q |
| VVMO6_01903 | L,D-Transpeptidase YcbB | 0.50 (0.012) | E |
| VVMO6_01909 | MoxR-like ATPase | 0.40 (0.033) | C |
| VVMO6_01912 | Oligopeptide transport ATP-binding protein OppD | 0.29 (0.006) | E |
| VVMO6_01913 | Dipeptide ABC transporter periplasmic protein | 0.35 (0.001) | E |
| VVMO6_01914 | Oligopeptide transport system permease OppB | 0.41 (0.001) | E |
| VVMO6_01915 | Oligopeptide transport system permease OppB | 0.47 (0.046) | E |
| VVMO6_02014 | Oligopeptide ABC transporter periplasmic oligopeptide-binding protein OppA | 0.40 (0.010) | E |
| VVMO6_02200 | Citrate synthase | 0.47 (0.017) | C |
| VVMO6_02203 | Pyridine nucleotide-disulfide oxidoreductase; NADH dehydrogenase | 0.47 (0.038) | F |
| VVMO6_02233 | Nitrogen regulatory protein P-II | 0.22 (0.040) | E |
| VVMO6_02452 | Isocitrate lyase | 0.38 (0.018) | C |
| VVMO6_02552 | Glutamate synthase (NADPH) large chain | 0.25 (0.001) | E |
| VVMO6_02555 | Glutamate synthase (NADPH) small chain | 0.46 (0.037) | E |
| VVMO6_02752 | NADH dehydrogenase subunit II-related protein | 0.42 (0.041) | C |
| VVMO6_02832 | Oligopeptide transport ATP-binding protein OppF | 0.43 (0.030) | E |
| VVMO6_02834 | Dipeptide ABC transporter periplasmic protein | 0.21 (0.015) | E |
| VVMO6_02835 | Oligopeptide transport system permease OppC | 0.40 (0.011) | E |
| VVMO6_02958 | tRNA 5-methylaminomethyl-2-thiouridine synthase TusA | 0.46 (0.006) | F |
| VVMO6_03095 | Sugar ABC transporter periplasmic protein | 0.47 (0.017) | G |
| VVMO6_03103 | Oligopeptide ABC transporterperiplasmicoligopeptide-binding protein OppA | 0.41 (0.002) | E |
| VVMO6_03104 | Dipeptide/oligopeptide/nickel ABC transporter permease | 0.35 (0.002) | E |
| VVMO6_03111 | beta-Glucanase | 0.39 (0.049) | G |
| VVMO6_03310 | Purine nucleoside phosphorylase | 0.45 (0.013) | F |
| VVMO6_03466 | Anaerobic glycerol-3-phosphate dehydrogenase subunit C | 0.41 (0.020) | C |
| VVMO6_03954 | 3-Hydroxyisobutyrate dehydrogenase | 0.48 (0.017) | I |
| VVMO6_03956 | 3-Hydroxyisobutyryl-CoA hydrolase | 0.50 (0.021) | I |
| VVMO6_04112 | Polyhydroxyalkanoic acid synthase | 0.37 (0.028) | I |
| VVMO6_04114 | 3-Ketoacyl-CoA thiolase/acetyl-CoA acetyltransferase | 0.34 (0.005) | I |
| VVMO6_04115 | Acetoacetyl-CoA reductase | 0.34 (0.033) | I |
| VVMO6_04174 | cobalamin adenosyltransferase | 0.33 (0.008) | Q |
| VVMO6_04378 | TRAP transporter solute receptor TAXI family | 0.49 (0.013) | P |
| VVMO6_00198 | C4-dicarboxylate transporter DcuA | 2.44 (0.034) | G |
| VVMO6_00422 | Fe(2+)-trafficking protein YggX | 2.22 (0.036) | P |
| VVMO6_00423 | A/G-specific adenine glycosylase | 2.10 (0.035) | F |
| VVMO6_00533 | Dihydrolipoamide acetyltransferase component of pyruvate dehydrogenase complex | 2.41 (0.032) | C |
| VVMO6_00543 | 2-Amino-4-hydroxy-6-hydroxymethyldihydropteridine pyrophosphokinase | 2.52 (0.041) | H |
| VVMO6_00897 | Lactoylglutathione lyase | 2.90 (0.019) | E |
| VVMO6_01032 | 4-Hydroxybenzoyl-CoA thioesterase family active site | 2.29 (0.021) | I |
| VVMO6_01134 | Serine transporter | 2.34 (0.021) | E |
| VVMO6_01833 | Manganese-dependent inorganic pyrophosphatase | 2.07 (0.017) | P |
| VVMO6_01854 | UDP-2,3-diacylglucosamine hydrolase | 2.33 (0.017) | G |
| VVMO6_01865 | Na^+^/H^+^ antiporter | 2.00 (0.015) | P |
| VVMO6_01940 | 3-Oxoacyl-(acyl-carrier-protein) synthase KASII | 2.55 (0.019) | I |
| VVMO6_02113 | Aspartate/tyrosine/aromatic aminotransferase | 2.32 (0.009) | E |
| VVMO6_02330 | Methionine ABC transporter ATP-binding protein | 2.22 (0.043) | E |
| VVMO6_02888 | Coproporphyrinogen III oxidase oxygen-independent | 2.24 (0.049) | H |
| VVMO6_02984 | Peptide ABC transporter ATP-binding protein | 2.13 (0.039) | E |
| VVMO6_03432 | 2-Aminoethylphosphonate ABC transporter periplasmic binding component | 2.87 (0.019) | P |
| VVMO6_03548 | Pyruvate/2-oxoglutarate dehydrogenase complex dihydrolipoamide dehydrogenase component | 2.37 (0.011) | C |
| VVMO6_03745 | Phosphotransferase system IIA component | 2.02 (0.004) | G |
| VVMO6_03867 | Nitrate ABC transporter ATP-binding protein | 2.07 (0.021) | P |
| VVMO6_04034 | 2-Keto-4-pentenoate hydratase | 2.10 (0.027) | Q |
| VVMO6_04206 | Isochorismatase of siderophore biosynthesis | 2.40 (0.028) | Q |
| VVMO6_04209 | Aryl carrier domain | 2.21 (0.011) | Q |
| VVMO6_04484 | 5-Keto-D-gluconate 5-reductase | 2.41 (0.011) | I |
| **Cellular processing and signaling** | | | |
| VVMO6_00107 | Sensory box/GGDEF family protein | 0.39 (0.013) | T |
| VVMO6_00386 | ATPase-like phosphate starvation-inducible protein PhoH | 0.48 (0.007) | T |
| VVMO6_00578 | Multidrug resistance protein | 0.43 (0.004) | V |
| VVMO6_00808 | Flagellin protein FlaD | 0.48 (0.031) | N |
| VVMO6_00964 | Serine protein kinase (prkA protein) P-loop containing | 0.45 (0.013) | T |
| VVMO6_01150 | Chemotaxis protein CheY | 0.37 (0.001) | T |
| VVMO6_01910 | Outer membrane receptor protein | 0.03 (0.041) | M |
| VVMO6_02070 | Two component response regulator | 0.50 (0.008) | T |
| VVMO6_02071 | Signal transduction histidine kinase | 0.38 (0.015) | T |
| VVMO6_02252 | Flagellin protein FlaD | 0.46 (0.034) | N |
| VVMO6_02611 | Methyl-accepting chemotaxis protein | 0.44 (0.009) | N |
| VVMO6_03030 | GGDEF family protein | 0.47 (0.003) | T |
| VVMO6_03072 | Glutathione S-transferase | 0.44 (0.018) | O |
| VVMO6_03288 | Toxin secretion ATP-binding protein | 0.47 (0.034) | V |
| VVMO6_03289 | Secretion protein, HlyD family | 0.48 (0.022) | V |
| VVMO6_03290 | Agglutination protein | 0.51 (0.021) | M |
| VVMO6_03291 | Membrane protein | 0.46 (0.009) | M |
| VVMO6_03819 | Membrane-fusion protein | 0.41 (0.046) | M |
| VVMO6_03896 | Rhs family protein | 0.49 (0.037) | M |
| VVMO6_00720 | Methyl-accepting chemotaxis protein | 2.21 (0.016) | N |
| VVMO6_01684 | Trypsin | 2.71 (0.036) | O |
| VVMO6_01777 | Glutaredoxin 1 | 2.09 (0.046) | O |
| VVMO6_01981 | Catalase | 2.03 (0.006) | O |
| VVMO6_02437 | Iron binding protein IscA for iron-sulfur cluster assembly | 2.53 (0.032) | O |
| VVMO6_02369 | Multidrug ABC transporter ATPase and permease | 4.15 (0.031) | V |
| VVMO6_03131 | N-Acetylglucosamine-regulated outer membrane porin | 2.17 (0.008) | M |
| VVMO6_03294 | Small-conductance mechanosensitive channel | 2.18 (0.022) | M |
| VVMO6_03332 | BatA | 2.14 (0.017) | V |
| VVMO6_03351 | Capsular polysaccharide synthesis enzyme CpsA sugar transferase | 2.47 (0.033) | M |
| VVMO6_03828 | Thioredoxin 2 | 2.14 (0.005) | O |
| **Information storage and processing** | | | |
| VVMO6_00196 | Transcriptional regulator LuxR family | 0.47 (0.006) | K |
| VVMO6_00620 | Deoxyribonuclease YjjV | 0.47 (0.019) | L |
| VVMO6_01114 | DNA-binding response regulator LuxR family | 0.36 (0.030) | K |
| VVMO6_01979 | Glycine cleavage system regulatory protein | 0.36 (0.021) | K |
| VVMO6_01985 | BarA-associated response regulator UvrY (GacA SirA) | 0.003 (0.047) | K |
| VVMO6_02821 | DNA repair protein RadC | 0.38 (0.001) | L |
| VVMO6_02853 | Transcription accessory protein (S1 RNA-binding domain) | 0.48 (0.022) | K |
| VVMO6_02956 | Transcriptional regulator | 0.46 (0.010) | K |
| VVMO6_03084 | DNA-binding response regulator | 0.44 (0.025) | K |
| VVMO6_03192 | DNA-binding HTH domain-containing protein | 0.50 (0.002) | K |
| VVMO6_00471 | Sigma factor RpoE regulatory protein RseC | 2.11 (0.002) | K |
| VVMO6_01597 | DNA-directed RNA polymerase | 2.48 (0.002) | K |
| VVMO6_02120 | DNA-binding protein HU-beta | 2.83 (0.038) | L |
| VVMO6_02499 | response regulator of the LytR/AlgR family | 2.46 (0.021) | K |
| VVMO6_02561 | Transcriptional regulator, LysR family | 2.52 (0.037) | K |
| VVMO6_02639 | Transcriptional regulator, LuxZ family | 2.20 (0.016) | K |
| VVMO6_02848 | ATP-dependent DNA helicase RecG | 2.10 (0.001) | L |
| VVMO6_04102 | Sialic acid utilization regulator RpiR family | 2.17 (0.045) | K |
| **Poorly characterized genes** | | | |
| VVMO6_00153 | Putative acyltransferase | 2.79 (0.036) | R |
| VVMO6_00261 | Hypothetical protein | 0.25 (0.045) | S |
| VVMO6_00619 | Hypothetical protein | 0.38 (0.007) | S |
| VVMO6_01060 | Hypothetical protein | 0.47 (0.020) | S |
| VVMO6_01063 | Hypothetical protein | 0.39 (0.022) | S |
| VVMO6_01080 | Hypothetical protein | 0.18 (0.010) | S |
| VVMO6_01081 | Hypothetical protein | 0.28 (0.037) | S |
| VVMO6_01088 | Hypothetical protein | 0.42 (0.011) | S |
| VVMO6_01148 | Transporter | 0.45 (0.002) | R |
| VVMO6_01169 | Hypothetical protein | 0.38 (0.038) | S |
| VVMO6_01393 | Putative acetyltransferase | 0.47 (0.027) | R |
| VVMO6_01435 | Hypothetical protein | 0.40 (0.015) | S |
| VVMO6_01438 | Hypothetical protein | 0.45 (0.017) | S |
| VVMO6_01728 | Hypothetical protein | 0.50 (0.007) | S |
| VVMO6_01729 | Putative metal-dependent hydrolase | 0.46 (0.009) | R |
| VVMO6_02050 | Hypothetical protein | 0.44 (0.006) | S |
| VVMO6_02089 | Hypothetical protein | 0.45 (0.031) | S |
| VVMO6_02179 | Hypothetical protein | 0.44 (0.013) | S |
| VVMO6_02232 | Hypothetical protein | 0.27 (0.038) | S |
| VVMO6_02338 | Decarboxylase family protein | 0.40 (0.041) | R |
| VVMO6_02536 | Hypothetical protein | 0.42 (0.038) | S |
| VVMO6_02915 | Hypothetical protein | 0.29 (0.007) | S |
| VVMO6_02957 | Hypothetical protein | 0.50 (0.037) | S |
| VVMO6_03013 | Hypothetical protein | 0.37 (0.014) | S |
| VVMO6_03309 | Hypothetical protein | 0.47 (0.001) | S |
| VVMO6_03345 | Exopolyphosphatase-related protein | 0.45 (0.014) | R |
| VVMO6_03463 | Hypothetical protein | 0.48 (0.006) | S |
| VVMO6_03482 | Hypothetical protein | 0.33 (0.036) | S |
| VVMO6_03606 | Hypothetical protein | 0.44 (0.001) | S |
| VVMO6_03895 | Hypothetical protein | 0.49 (0.017) | S |
| VVMO6_04021 | Hypothetical protein | 0.33 (0.006) | S |
| VVMO6_04100 | Sialic acid-induced transmembrane protein YjhT | 0.39 (0.027) | S |
| VVMO6_04283 | hypothetical protein | 0.28 (0.048) | S |
| VVMO6_04381 | hypothetical protein | 0.43 (0.042) | S |
| VVMO6_00122 | hypothetical protein | 3.40 (0.015) | S |
| VVMO6_00316 | hypothetical protein | 2.10 (0.034) | S |
| VVMO6_00682 | DNA damage-inducible protein in SOS regulon dependent on cyclic AMP and H-NS | 2.56 (0.038) | R |
| VVMO6_01159 | Hypothetical protein | 2.19 (0.019) | S |
| VVMO6_01588 | Hypothetical protein | 2.48 (0.049) | S |
| VVMO6_01594 | hypothetical protein | 2.88 (0.048) | S |
| VVMO6_01950 | hypothetical protein | 2.14 (0.027) | S |
| VVMO6_01992 | hypothetical protein | 2.09 (0.002) | S |
| VVMO6_02015 | hypothetical protein | 2.26 (0.034) | S |
| VVMO6_02084 | hypothetical protein | 2.44 (0.028) | S |
| VVMO6_02627 | transamidase GatB domain-containing protein | 3.57 (0.004) | R |
| VVMO6_03028 | phage terminase large subunit | 2.25 (0.033) | R |
| VVMO6_03228 | hypothetical protein | 2.40 (0.042) | S |
| VVMO6_03811 | hypothetical protein | 2.11 (0.002) | S |

^a^Normalized transcript level in the *ΔvarA* mutant to the wild type as determined by microarray analysis. The presented data include the normalized values <0.5 or >2 with P <0.05.

^b^COGs: K, transcription; L, replication, recombination and repair; V. defense mechanisms; T, signal transduction mechanisms; M, cell wall/membrane/envelope biogenesis; N, cell motility; O, posttranslational modification, protein turnover, and chaperones; C, energy production and conversion; G, carbohydrate transport and metabolism; E, amino acid transport and metabolism; F, nucleotide transport and metabolism; H, coenzyme transport and metabolism; I, lipid transport and metabolism; P, inorganic ion transport and metabolism; Q, secondary metabolite biosynthesis, transport and catabolism; R, general function prediction only; S, function unknown.
