# Supplementary material for: Role of AcsR in expression of the acetyl-CoA synthetase gene in Vibrio vulnificus
Source: BMC Microbiol. 2015 Apr 12;15:86. doi: 10.1186/s12866-015-0418-4 (PMC4409781; doi:10.1186/s12866-015-0418-4)
Supplement: Additional file 2: Figure S1. — Construction of ΔacsS mutant V. vulnificus. A - Construction of V. vulnificus mutant defective in acsS by using two sets of primers (indicated by horizontal arrows with the primer names listed in Additional file 4: Table S2) to delete VVMO6_00191. A bar represents the length of DNA equivalent to 500 bp; B - Deletion of the corresponding gene was examined by PCR using a pair of primers, acsSupF and acsSdownR. SM indicates DNA size markers. [file 12866_2015_418_MOESM2_ESM.pptx]

## Slide 1
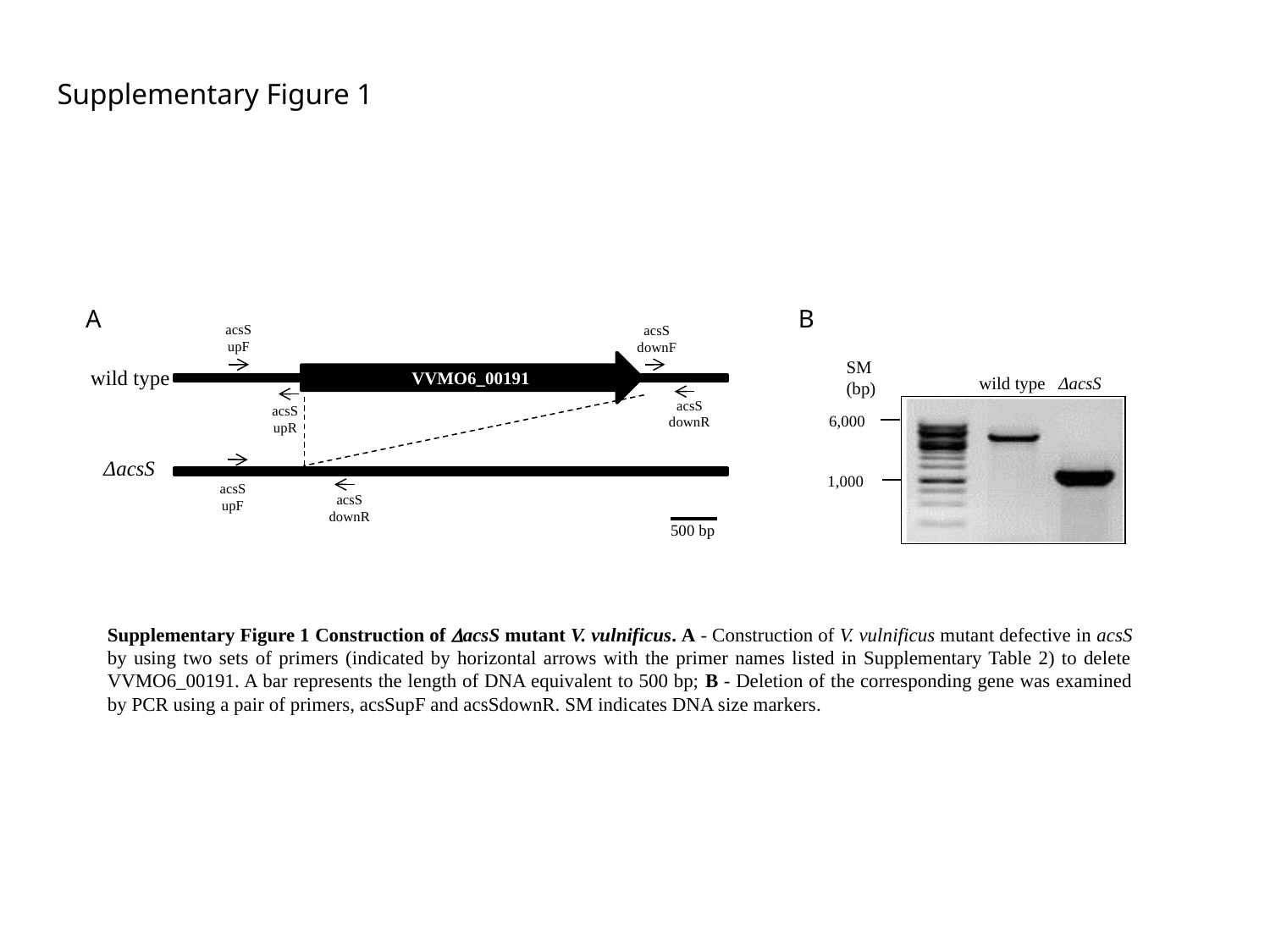

Supplementary Figure 1
A
B
acsS
upF
acsS
downF
wild type
VVMO6_00191
acsS
downR
acsS
upR
ΔacsS
acsS
upF
acsS
downR
500 bp
SM
(bp)
wild type
ΔacsS
6,000
1,000
Supplementary Figure 1 Construction of ΔacsS mutant V. vulnificus. A - Construction of V. vulnificus mutant defective in acsS by using two sets of primers (indicated by horizontal arrows with the primer names listed in Supplementary Table 2) to delete VVMO6_00191. A bar represents the length of DNA equivalent to 500 bp; B - Deletion of the corresponding gene was examined by PCR using a pair of primers, acsSupF and acsSdownR. SM indicates DNA size markers.
