# Supplementary material for: Role of AcsR in expression of the acetyl-CoA synthetase gene in Vibrio vulnificus
Source: BMC Microbiol. 2015 Apr 12;15:86. doi: 10.1186/s12866-015-0418-4 (PMC4409781; doi:10.1186/s12866-015-0418-4)
Supplement: Additional file 3: Figure S2. — Construction of ΔacsA mutant V. vulnificus. A - Construction of V. vulnificus mutant defective in acsA by using two sets of primers (indicated by horizontal arrows with the primer names listed in Additional file 4: Table S2) to delete VVMO6_00187. A bar represents the length of DNA equivalent to 500 bp; B - Deletion of the corresponding gene was examined by PCR using a pair of primers, acsAupF and acsAdownR. SM indicates DNA size markers. [file 12866_2015_418_MOESM3_ESM.pptx]

## Slide 1
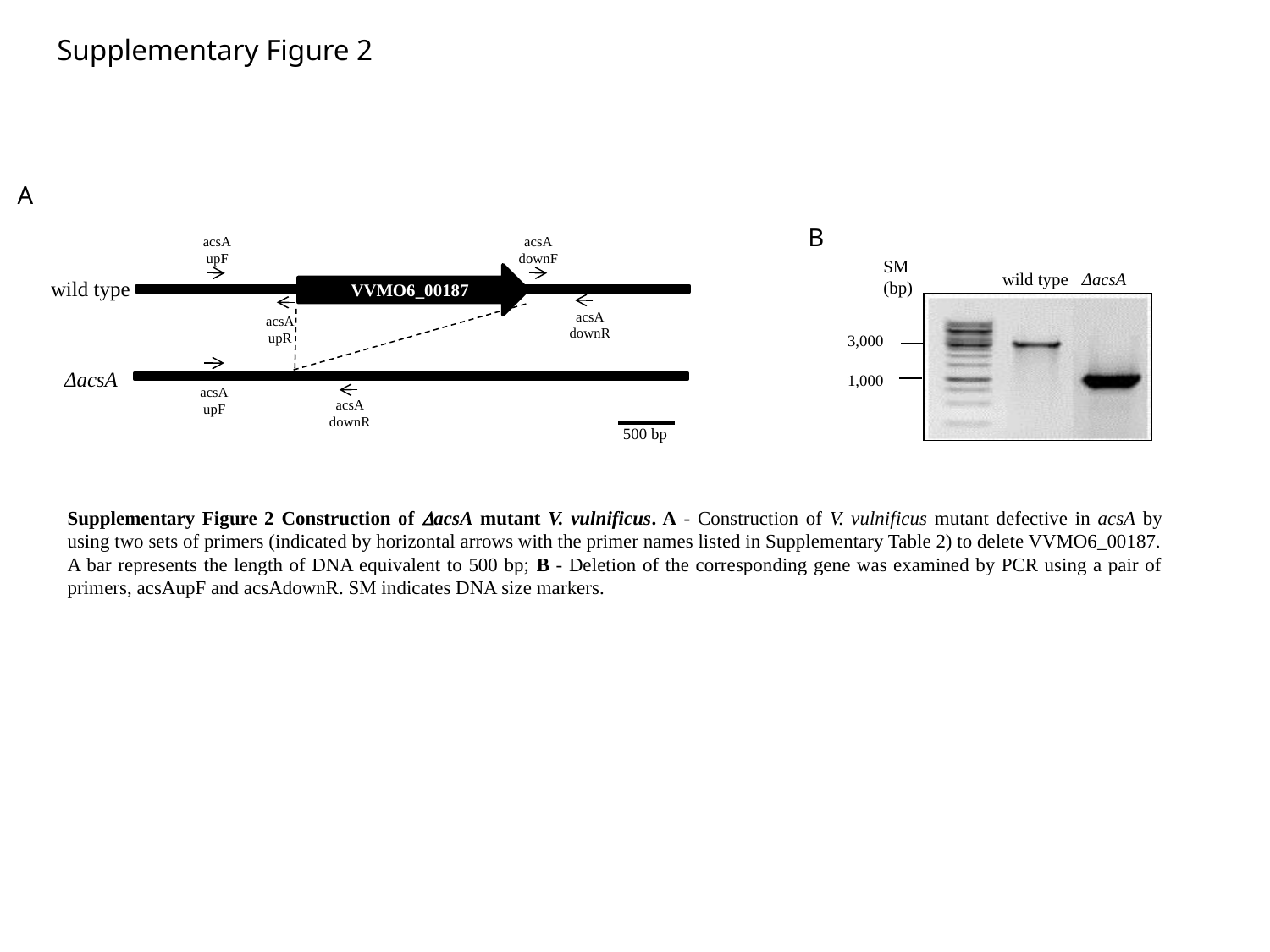

Supplementary Figure 2
A
B
acsA
upF
acsA
downF
wild type
VVMO6_00187
acsA
downR
acsA
upR
ΔacsA
acsA
upF
acsA
downR
500 bp
SM
(bp)
wild type
ΔacsA
3,000
1,000
Supplementary Figure 2 Construction of ΔacsA mutant V. vulnificus. A - Construction of V. vulnificus mutant defective in acsA by using two sets of primers (indicated by horizontal arrows with the primer names listed in Supplementary Table 2) to delete VVMO6_00187. A bar represents the length of DNA equivalent to 500 bp; B - Deletion of the corresponding gene was examined by PCR using a pair of primers, acsAupF and acsAdownR. SM indicates DNA size markers.
