# Supplementary material for: Role of AcsR in expression of the acetyl-CoA synthetase gene in Vibrio vulnificus
Source: BMC Microbiol. 2015 Apr 12;15:86. doi: 10.1186/s12866-015-0418-4 (PMC4409781; doi:10.1186/s12866-015-0418-4)
Supplement: Additional file 4: Table S2. — Oligonucleotide primers used in this study. [file 12866_2015_418_MOESM4_ESM.docx]

**Supplementary Table 2 Oligonucleotide primers used in this study**

| Gene | Primer | | Nucleotide sequences (5'→3') ^a^ |
| --- | --- | --- | --- |
| For construction of *V. vulnificus* mutants | | | |
| *varA* | varAupF | GATCGTCGACTTGCTAAGCGTTGGGCATCTTC | |
|  | varAupR | GATCCTGCAGGATCAAACTTGTATCTCCACAC | |
|  | varAdownF | GATCCTGCAGACCGAGACTCTCTAGTGTGAC | |
|  | varAdownR | GATCTCTAGAACATCAAACAAGGACGCGTTCG | |
| *acsR* | luxRupF | CATGGGGCCCGACCCAGCCGTGGCAGCTCAAC | |
|  | luxRupR | CATGCTGCAGCATGACGTTAAACACTTTTAAC | |
|  | luxRdownF | CATGCTGCAGCAATAACTCAACGCCAAACTC | |
|  | luxRdownR | CATGGAGCTCGAAAATCATACTCAGTGCGAA | |
| *acsS* | acsSupF | ATGCCTCGAGAGTGAAAGACCGAAGCTG | |
|  | acsSupR | ATGCGGATCCGAGACATCCTTTCTCTTG | |
|  | acsSdownF | ATGCGGATCCGGTGCCTTCTCCAAAAAG | |
|  | acsSdownR | ATGCTCTAGAATTCTCTACGCATACCATG | |
| *acsA* | acsAupF | ATGCCTCGACAACAAAGAGTATGTCTCTC | |
|  | acsAupR | ATGCGGATCCTATCTCTCTCCTTGTGCC | |
|  | acsAdownF | ATGCGGATCCTCCGTTTTAAACAAAACACC | |
|  | acsAdownR | ATGCTCTAGATGATGATGATGAAGTCGAC | |
| For construction of complementation plasmids | | | |
| *acsR* | acsRcomF | CATGAAGCTTCCAACAAGTGCTCGGCCTACC | |
|  | acsRcomR | CATGGGATCCTTATTGCTCGGGCTCTAGC | |
| *acsS* | acsScomF | ATGCCTGCAGATGCAAGGATGGTTGGTG | |
|  | acsScomR | ATGCGGATCCTCACGCCACTCGGTTCAA | |
| *acsA* | acsAcomF | ATGCAAGCTTCAAGACAAACACGATGCG | |
|  | acsAcomR | ATGCGGATCCTTAAACCAGTTCGGCTTT | |
| For construction of an *acsA* transcriptional fusion (pHKacsA::luxAB) | | | |
| *acsA* | acsAproF | ATGCGGTACCCAAGACAAACACGATGCG | |
|  | acsAproR | ATGCGGATCCACTCATTGTCTCTCTCCT | |
| For overexpression of the recombinant protein AcsR (pETacsR) | | | |
| *acsR* | racsRF | CATGGGATCCGATGGATTCGACCTACACCAT | |
|  | racsRR | CATGAAGCTTTTATTGCTCGGGCTCTAGC | |
| For real-time PCR | | | |
| *acsR* | acsR-F | GGCCGAGAAAATCGCCGCGT | |
|  | acsR-R | TTGCTCGGCTCTAGCTGCT | |
| *acsA* | acsA-F | TACGCCTACGTCACCCTCAAT | |
|  | acsA-R | GGTGTCACCCAAGTTGCTGGT | |
| *gap* | GAPDH-F | GCATTCTCGGCTTTGAAGAG | |
|  | GAPDH-R | GCCCATTTCGTTGTCATACC | |

^a^Restriction enzyme sites, if any, are underlined.
